# Characteristics, health risks, and premature mortality attributable to ambient air pollutants in four functional areas in Jining, China

**DOI:** 10.3389/fpubh.2023.1075262

**Published:** 2023-01-19

**Authors:** Yue Yuan, Xi Zhang, Jingfeng Zhao, Fuzhen Shen, Dongyang Nie, Bing Wang, Lei Wang, Mengyue Xing, Michaela I. Hegglin

**Affiliations:** ^1^Jining Meteorological Bureau, Shandong, China; ^2^Institute of Energy and Climate Research, IEK-7: Stratosphere, Forschungszentrum Jülich, Jülich, Germany; ^3^Department of Meteorology, University of Reading, Reading, United Kingdom; ^4^School of Environmental Science and Engineering, Southern University of Science and Technology, Shenzhen, China; ^5^Henley Business School, University of Reading, Reading, United Kingdom; ^6^Jining Bureau of Ecology and Environment, Shandong, China; ^7^Business School, Dalian University of Foreign Languages, Liaoning, China

**Keywords:** air pollution, functional regions, health effect, potential source, premature mortality

## Abstract

Air pollution is one of the leading causes for global deaths and understanding pollutant emission sources is key to successful mitigation policies. Air quality data in the urban, suburban, industrial, and rural areas (UA, SA, IA, and RA) of Jining, Shandong Province in China, were collected to compare the characteristics and associated health risks. The average concentrations of PM_2.5_, PM_10_, SO_2_, NO_2_, and CO show differences of −3.87, −16.67, −19.24, −15.74, and −8.37% between 2017 and 2018. On the contrary, O_3_ concentrations increased by 4.50%. The four functional areas exhibited the same seasonal variations and diurnal patterns in air pollutants, with the highest exposure excess risks (ERs) resulting from O_3_. More frequent ER days occurred within the 25–30°C, but much larger ERs are found within the 0–5°C temperature range, attributed to higher O_3_ pollution in summer and more severe PM pollution in winter. The premature deaths attributable to six air pollutants can be calculated in 2017 and 2018, respectively. Investigations on the potential source show that the ER of O_3_ (*r* of 0.86) had the tightest association with the total ER. The bivariate polar plots indicated that the highest health-based air quality index (HAQI) in IA influences the HAQI in UA and SA by pollution transport, and thus can be regarded as the major pollutant emission source in Jining. The above results indicate that urgent measures should be taken to reduce O_3_ pollution taking into account the characteristics of the prevalent ozone formation regime, especially in IA in Jining.

## 1. Introduction

Air pollution has attracted significant concern worldwide in recent decades, especially in China due to the highest ranking of death records across the world (1). Many previous studies have reported that exposure to both ambient and indoor air pollutants has a direct association with a significantly increased risk of cardiovascular, respiratory, and coronary heart diseases, and even can induce cancer (2–7). Moreover, numerous studies have demonstrated that no matter the long-term or short-term exposure, the varied risk and non-accident premature mortality could be attributed to exposure levels of different air pollutants [i.e., particulate matter with an aerodynamic diameter < 2.5 and 10 μm (PM_2.5_ and PM_10_), nitrogen dioxide (NO_2_), sulfur dioxide (SO_2_), ozone (O_3_), carbon monoxide (CO)] in one city or at the national scale (8–18).

Health impacts from different air pollutants are usually assessed by epidemiology, toxicology and clinical studies (19, 20). One of the popularly used approaches is the epidemiological statistics method, which can be used to calculate the coefficient of the exposure-response relationship based on the relative mortality risk of air pollutants (21), thus linking pollutants with health risks. At present, many health impact assessment studies have investigated the health risks or premature mortality attributable to a single air pollutant or adjusted for exposure to other pollutants globally or regionally (22–27). In China, numerous epidemiological literature concentrated on the association of single pollutants and population health has been designed by using various methods, which include time-series, cross-sectional, panel, case-crossover, cohort and intervention designs (28). To make an assessment of the short-term health effects of one single air pollutant, time-series studies coupled with Poisson regression or Generalized Additive Model (GAM) were conducted to explore the association of different air pollutants [like NO_2_ (29), CO (30), SO_2_ (31), PM_10_ (32), PM_2.5_ (33), and O_3_ (34)] and daily mortality in large Chinese cities, including Beijing, Shanghai, Chongqing, Shenyang, and Wuhan (28). Because of the easier conducted research experiment and clearly interpretable result, single-pollutant air quality strategies are widely applicable to protect human health for policy-makers (35). However, the health effect of single-pollutant should be applied cautiously. Because of the certain correlation among different air pollutants, identifying the independent effects of single-pollutant become much more difficult (36). Moreover, the air that humans breathe at once is multiple pollutants. Therefore, exploring the joint effect associated with multi-pollutant should be taken into consideration urgently by scholars.

Currently, three typical approaches, including statistical regression models, the indicator approach, and the source identification methods, can be used to quantify the joint health risk from multi-pollutant (35). Generally, the indicator approach means that it is to use one pollutant to represent the total exposure to several pollutants. To evaluate the total health risks and premature mortalities attributed to different air pollutants (here including PM_2.5_, PM_10_, SO_2_, NO_2_, O_3_, and CO), how to select an appropriate pollutant or construct a health risk index has become more significant. Currently, the air quality is characterized by the widely used Air Quality Index (AQI) system, an index implemented by the central government (like in the US or China) is determined by the primary pollutant rather than the overall air condition (37). To address the inadequacy of the single-pollutant-oriented AQI, the aggregate AQI (AAQI) (38) and air quality health index (AQHI) (39) have been developed and applied in practice. In a recent study, Hu et al. (40) using a novel index referred to as the health risk-based AQI (HAQI), investigated air quality in six representative Chinese cities and found that the total days in a given AQI category (either unhealthy or very unhealthy) were including days in HAQI categories that were equal or even higher than the respective AQI category (i.e., very unhealthy or hazardous). Shen et al. (41) applied the HAQI in 367 cities in China, showing high HAQI to be most prevalent in the North China Plain region (NCP). Zhou et al. (42) established the HAQI in 366 cities in China and found organics were driving PM_2.5_-formation when PM_2.5_ is at a lower level of health risk.

Here, we expand on these studies, which focused on atmospheric pollution at the city level (that is averaged over whole cities), to investigate multi-pollutant exposure health risks associated with different functional areas within a city. To this end, we applied the HAQI calculation to observations obtained from four functional areas in Jining city. Meanwhile, to identify which functional areas and air pollutants play the dominant role in Jining, we introduced the potential source contribution function (PSCF) model in this study as well. The PSCF is a conditional probability model by coupling the pollutant with an air mass arriving at the observational site after having passed through a specific geographical area (43). The PSCF value is determined by dividing the space up into certain grid cells and checking the back-trajectory endpoint to see if there was a sampling day commensurate with the trajectory. The PSCF analysis is widely applied to identify the potential source of any pollutant, like SO_2_ (44), NO_2_ (45), PM_2.5_ (46), PM_10_ (47), CO (48), and O_3_ (48) black carbon particles (49), or a pollutant-related indicator (e.g., excess risk in section 2.4) (41).

At last, the results aim at providing a clear understanding of the regional distribution of health risks and to provide guidance to policy-makers for effective mitigation policies within Jining's city borders. In particular, Jining is located between the Beijing-Tianjin-Hebei region and the Yangtze River Delta, which is prone to air pollution under a zonal circulation and stable synoptic conditions (low wind and high relative humidity) aside from strong emissions of pollutants, especially in winter and spring. To evaluate the air quality expected over the 2017–2018 period and the associated feedback on health risks in four different functional regions in Jining city, this study aims to: (1) compare the air pollution levels across the four functional areas; (2) estimate the multi-pollutant exposure health risk in these functional areas; (3) evaluate all-cause premature mortalities attributable to all air pollutants, and (4) identify which functional areas and air pollutants are the major contributors to the health risk of Jining City.

## 2. Materials and methods

### 2.1. Site and data

**Figure 1:**
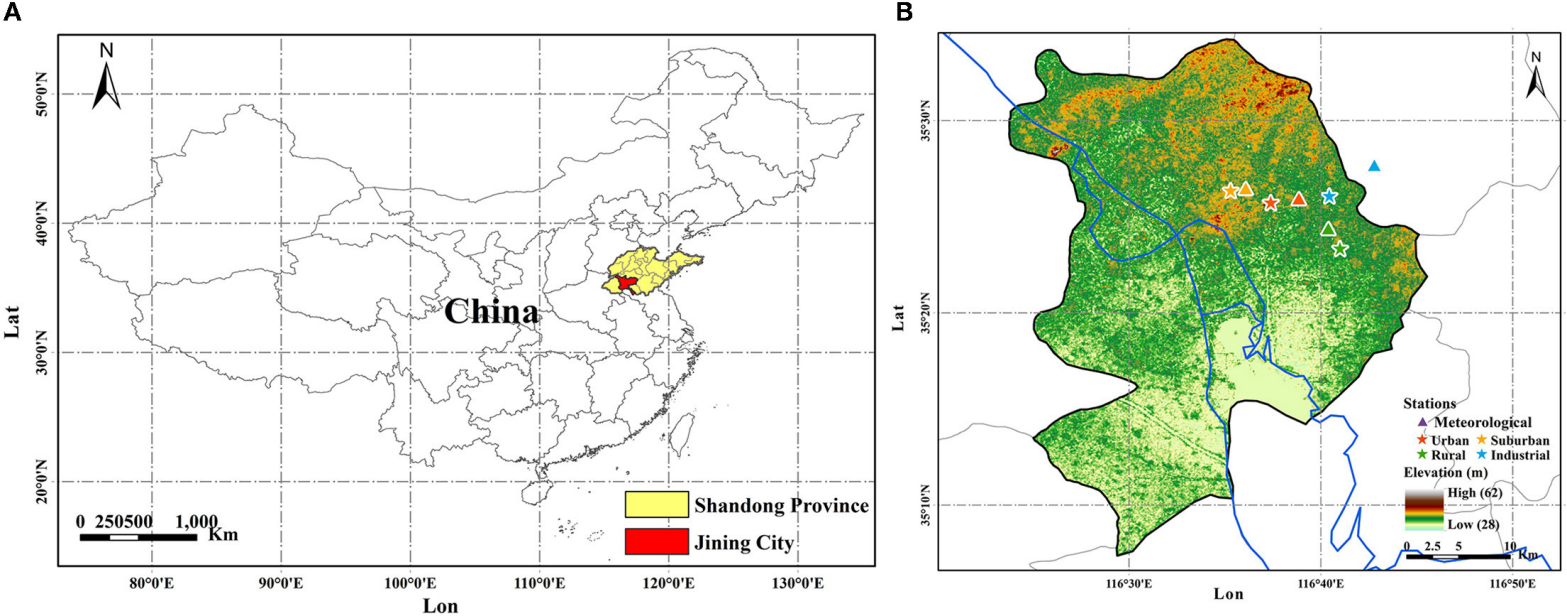
The location of Jining in Shandong Province **(A)**, and the meteorology and air quality stations **(B)** in Jining city. Colored stars and triangles represent air quality sites and meteorological site in four functional areas (Suburban: orange, Urban: red, Rural: green, Industry: blue).

The study region, Jining (116°26′-116°44′E, 35°08′-35°32′N), is located in the southwest of Shandong Province in eastern China (Figure 1A). The air sampling sites (colored stars) and meteorological stations (colored triangles) are located in the four different functional areas identified in Jining. Highways and industrial parks are found near the industrial (IA) site. For the urban (UA) site in the city center, the nearby road network is complex, with heavy traffic and high building density. The suburban (SA) site is located between the urban and rural areas, and the rural (RA) site is located by a farm and river and far away from the city center. The surrounding environment of each air quality sampling site is largely consistent with the basic characteristics of the functional areas. The meteorological stations were chosen to be as close as possible to the air pollutant sampling sites.

The hourly monitoring data of six pollutants and the hourly meteorological data at each site were obtained from the website of the Environmental Meteorological Platform of Shandong (http://10.76.10.119/) and the Jining Meteorological Bureau, respectively. The meteorology factors include temperature (°C), wind direction (WD), and wind speed (WS). Here, WS and WD were used to explore the potential source region of pollution. The temperature was applied to investigate the impact on air pollutants, especially for O_3_. Based on the daily minimum requirement for the validity of air pollutant concentration data (Chinese Ambient Air Quality Standard GB 3095-2012) (https://www.mee.gov.cn/ywgz/fgbz/bz/bzwb/dqhjbh/dqhjzlbz/201203/t20120302_224165.shtml), the daily and monthly data during 2017 (2018) reported in this study are valid for 362 (365) days and 12 (12) months, respectively. The other days in 1 year were deleted due to the sampling data being < 20 h in a day. For the meteorology dataset, all the data used each day is valid according to China's Surface Meteorological Observation Standard (CSMOS) (https://www.cma.gov.cn/zfxxgk/gknr/flfgbz/bz/202209/t20220921_5099079.html). For precipitation and relative humidity, we did not explore the impact of the two meteorology factors on air pollution due to the large number of missing values.

### 2.2. The calculation method of excess risk and health-risk based AQI

The relative risk (RR) of each pollutant is expressed by an exponential-linear function as shown in Eq. 1 (40). Here, β_i_ is the exposure-response relationship coefficient (which quantifies the additional health risk per unit increase of an air pollutant) with values of 0.038, 0.032, 0.081, 0.13, and 0.048% per μg/m^3^ for PM_2.5_, PM_10_, SO_2_, NO_2_, and O_3_, respectively, and 3.7% per mg/m^3^ for CO (50). C_*i*_ represents the mass concentration of a pollutant *i*. Meanwhile, a baseline concentration C_i,0_ is also defined to determine the minimum risk of each pollutant *i*, meaning one pollutant has no health risk when its concentration is below or equal to C_0_, that is, RR_i_ = 1. Here, the upper threshold values of Chinese Ambient Air Quality Standard (CAAQS) 24-h Grade II were regarded as the C_*i*,0_ (Supplementary Table 1). The excess risk (ER) of pollutant *i* is written as in Eq. 2 and the total ER can be calculated by adding up the ER of each pollutant (Eq. 3). It should be noted that the ER added up linearly could over-estimate the assessment of total ER if those pollutants are highly correlated. Therefore, the total ER from six air pollutants can be regarded as an upper-bound estimation (40).

(1) $$\begin{array}{l} RR_{i}=exp\left[\beta_{i}\left(C_{i}-C_{i,0}\right)\right],\;C_{i}>C_{i,0} \end{array}$$

(2) $$\begin{array}{l} ER_{i}=RR_{i}-1 \end{array}$$

(3) $$\begin{array}{l} ER_{total}=\overset{n}{\underset{i=1}{\sum }}ER_{i}=\overset{n}{\underset{i=1}{\sum }}\left(RR_{i}-1\right). \end{array}$$

After calculating the total ER, the combined multi-pollutant Relative Risk (RR^*^) and an equivalent total concentration (${C_{i}}^{*}$) of pollutant *i* (40) can be written as:

(4) $$\begin{array}{l} {RR}^{*}=ER_{total}+1=exp\left[\beta \left(C^{*}-C_{0}\right)\right] \end{array}$$

(5) $$\begin{array}{l} {C_{i}}^{*}=\frac{ln\left(RR^{*}\right)}{\beta_{i}+C_{0,i}.} \end{array}$$

Finally, ${C_{i}}^{*}$ is substituted for the *C*_*i,m*_ in the AQI calculation to yield the HAQI (40), where the AQI calculation is as follows:

(6) $$\begin{array}{l} AQI_{i}=\frac{AQI_{i,j}-AQI_{i,j-1}}{\left(C_{i,j}-C_{i,j-1}\right)}\times \left(C_{i,m}-C_{i,j-1}\right)+AQI_{i,j-1},\;j>1 \end{array}$$

(7) $$\begin{array}{l} AQI_{i}=AQI_{i,1}\frac{C_{i,m}}{C_{i,1}},\;j=1 \end{array}$$

(8) $$\begin{array}{l} AQI=max\left(AQI_{1},AQI_{2}\ldots ,AQI_{n}\right),\;n=1,\;2,\;\ldots ,\;6. \end{array}$$

where *C*_*i,m*_ is the measured concentration of pollutant *i*; *j* is the health category index; *C*_*i,j*_ is the reference concentration for pollution *i* corresponding to the *j-*th health category. Accordingly, the HAQI calculation could be demonstrated as follows:

(9) $$\begin{array}{l} HAQI_{i}=\frac{HAQI_{i,j}-HAQI_{i,j-1}}{\left(C_{i,j}-C_{i,j-1}\right)}\times \left({C_{i}}^{*}-C_{i,j-1}\right)+\\ \;HAQI_{i,j-1},\;j>1, \end{array}$$

(10) $$\begin{array}{l} HAQI_{i}=HAQI_{i,1}\frac{{C_{i}}^{*}}{C_{i,1}},\;j=1 \end{array}$$

(11) $$\begin{array}{l} HAQI=max\left(HAQI_{1},HAQI_{2}\ldots ,HAQI_{n}\right),\;n=1,\;2,\;\ldots ,\;6. \end{array}$$

### 2.3. Daily cause-specific mortality and health burden assessment

The annual all-cause mortality in Jining was obtained from the Jining Statistical Yearbooks 2017 and 2018. The daily mortality was then calculated by the annual mortality rate divided by the number of days per year. The estimated health burden owing to short-term exposure to air pollutants can be calculated as follows (51, 52):

(12) $$\begin{array}{l} M=\overset{n}{\underset{i}{\sum }}AF_{i}\times BM \end{array}$$

(13) $$\begin{array}{l} AF_{i}=\left(RR_{i}-1\right)/RR_{i} \end{array}$$

where *M* (total mortality due to atmospheric pollution), *n* (total number of days), BM (daily baseline mortality), *AF*_*i*_ (daily attributable fraction related to short-term exposure of air pollutant *i*).

### 2.4. Potential source contribution function analysis

In this study, back trajectory analyses were performed by using the Hybrid Single-Particle Lagrangian Integrated Trajectory HYSPLIT model (Version 4.9) (53). The 72 h back trajectories arriving at Jining city at a height of 300 m were calculated every 3-h from 2017 to 2018. Based on these back trajectories data, a potential source contribution function (PSCF) analysis (54) was executed with ZeFir, an Igor-based (Wavemetrics, USA) package (55). PSCF analyses are commonly used to investigate the origin of observed concentrations at a sampling site under a given criterion (here, the 75th percentile value).

(14) $$\begin{array}{l} \;PSCF_{i,j}=\frac{m_{i,j}}{n_{i,j}} \end{array}$$

where *n*_*i,j*_ and *m*_*i,j*_ are the total count of endpoints and above-threshold endpoints located in the *i, jth* air cell, respectively. A sigmoid weighting function (41) was used to reduce the influence of large differences between two air cells (see Eq. 15). Three values in this function are 10, 0.5, 0.1 for *a, b, c* respectively (41). It is written as follows:

(15) $$\begin{array}{l} W=\frac{1}{\left(1+c\right)\left(1+e^{-a\left(x-b\right)}\right)}+\frac{c}{1+c} \end{array}$$

(16) $$\begin{array}{l} x=\log\left(n_{i,j}+1\right)/{\text{max}}_{log\left(n_{i,j}+1\right)} \end{array}$$

After calculating the PSCF for each sampling site in one city individually, the combined PSCF over all the sampling sites in the city can be calculated by using a multi-site (MS) merging method:

(17) $$\begin{array}{l} MS_{i,j}=\;\frac{\sum_{l}m_{i,j}^{l}}{\sum_{l}n_{i,j}^{l}} \end{array}$$

where *m*^l^ and *n*^l^ values indicate the *m* and *n* number counts of the sampling sites *l* in Jining.

## 3. Results and discussion

### 3.1. Comparison of six pollutants in four functional areas

**Figure 2:**
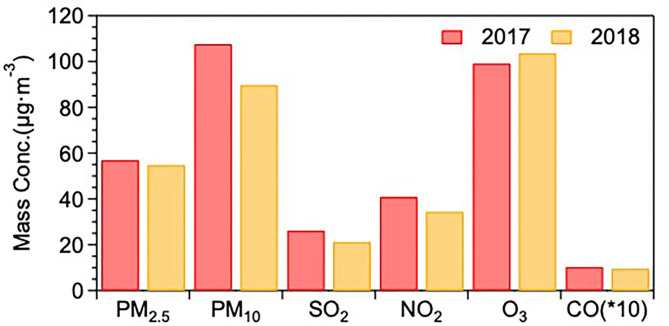
The annual mean mass concentrations of six pollutants in Jining during 2017 and 2018 [the unit of CO is mg/m^3^, CO (*10) means the real CO mass concentration multiple 10].

Figure 2 shows the annual mean mass concentrations of six pollutants in Jining during 2017 and 2018 at the city level. PM_2.5_, PM_10_, SO_2_, NO_2_, and CO all show lower values in 2018 than in 2017, indicating decreased emissions between the 2 years with 3.87% (from 57.11 to 54.89 μg/m^3^), 16.67% (from 107.65 to 89.71 μg/m^3^), 19.24% (from 26.27 to 21.21 μg/m^3^), 15.74% (from 40.97 to 34.52 μg/m^3^) and 8.37% (from 10.43 to 9.56 mg/m^3^), respectively. Conversely, the mass concentration of O_3_ was elevated by 4.5% (from 99.26 μg/m^3^ to 103.72 μg/m^3^). Elevated O_3_ mass concentrations and decreased mass loadings of PM have become a generally observed phenomenon resulting from pollution control measures, indicating that fewer PM but more O_3_ pollution events may also occur in Jining city in the future. Following many previous studies reports (56–59), this finding also stresses the key role of controlling O_3_ pollution through a series of strategies, such as the reduction of anthropogenic emissions, adjustment of the temperature, and balanced NOx and VOC control, for the local government in the future.

**Figure 3:**
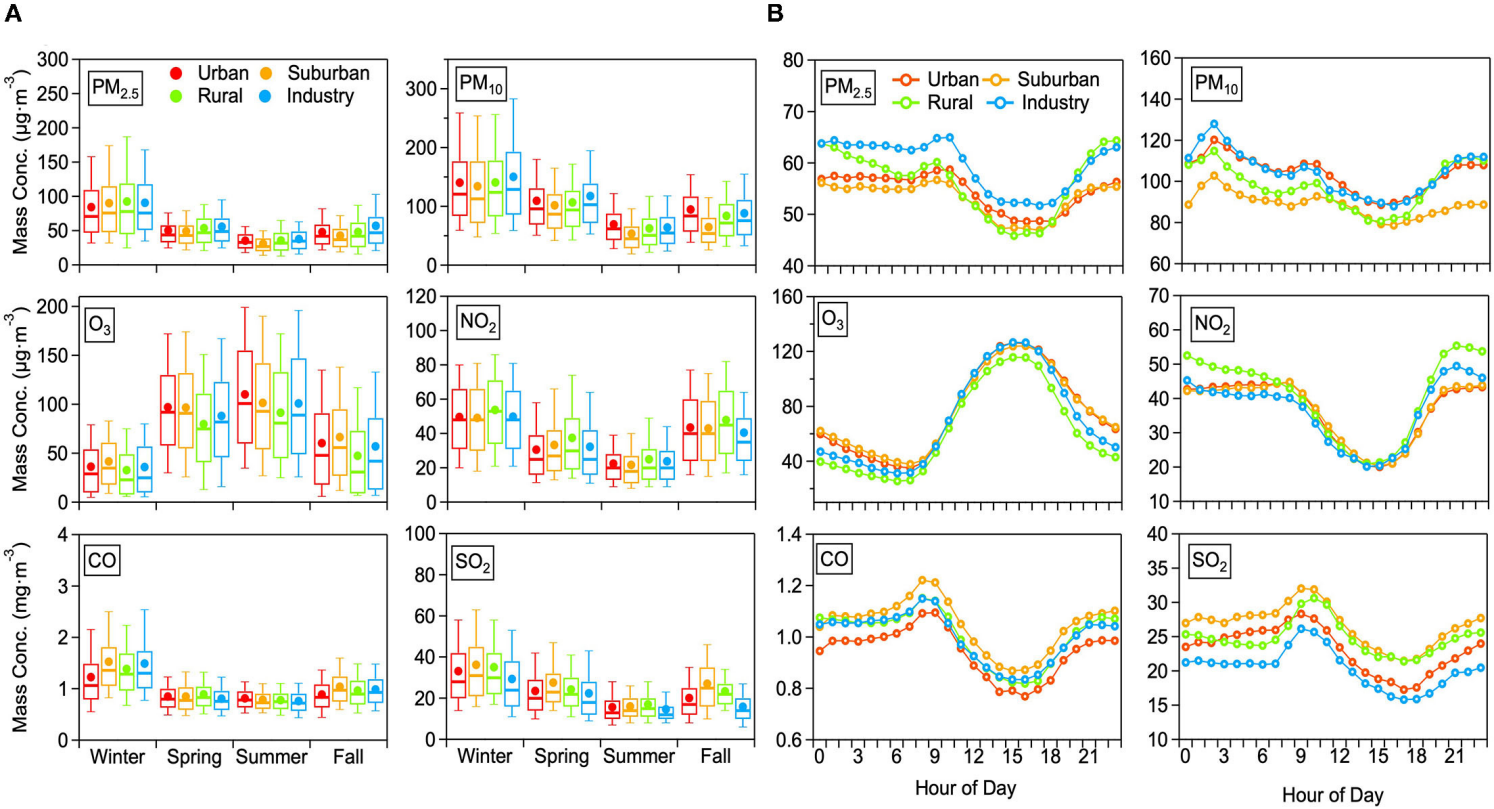
The seasonal **(A)** and diurnal **(B)** distributions of the six pollutants averaged over 2017 and 2018 for each of the functional areas (color-coded).

The seasonal distributions of the six pollutants averaged over the 2 years were then compared among four functional areas: UA, SA, RA, and IA, with the results shown in Figure 3A. Overall, the mass loading of all pollutants (except for O_3_) exhibited high (low) mass concentrations in winter and low (high) mass concentrations in summer. The seasonal patterns of all the air pollutants' mass loadings in Jining are consistent with that in almost all other cities across China (41, 42). The higher concentrations of the six air pollutants, except for ozone, in winter, can be explained by enhanced coal combustion, biomass burning, and unfavorable meteorological conditions, including low temperature (2.6°C), and boundary layer height (395 m) in winter (Supplementary Figure 1). The opposite behavior of the ozone concentrations, with the highest values during spring/summer is a well-known consequence of photochemistry, which is most active in these seasons.

After identifying the seasonal patterns of the six air pollutants in the four functional areas, the differences in the annual mean behavior (averaged over 2017 and 2018) of the mass loadings of the six air pollutants among UA, SA, RA, and IA are discussed. For PM (PM_10_ and PM_2.5_), the order of mass loading from high to low follows as: IA (105.00 and 60.37 μg/m^3^) > RA (103.57 and 57.56 μg/m^3^) > UA (98.52 and 54.49 μg/m^3^) > SA (88.61 and 53.26 μg/m^3^). With the contribution of fossil fuel combustion from plenty of power plants and the emissions from factories in this area, the IA had a higher mass concentration of PM than in the other three areas in all four seasons (except for the PM_10_ in summer and fall). The local source of high mass loading of PM in RA results mainly from residents cooking and straw burning. The higher PM_10_ in UA compared to that in IA in summer and fall might be ascribed to the heavy traffic emissions and unfavorable pollution dilution conditions due to high building density. For NO_2_ and SO_2_, the mass concentrations in these areas followed the order of RA (41.02 μg/m^3^) > UA (36.79 μg/m^3^) > IA (36.61 μg/m^3^) > SA (36.52 μg/m^3^) and SA (26.74 μg/m^3^) > RA (24.92 μg/m^3^) > UA (23.13 μg/m^3^) > IA (20.56 μg/m^3^), respectively. The mass concentrations of NO_2_ and SO_2_ were the highest in IA and SA, respectively. For CO, the mass loading was very similar during spring, summer, and fall. In winter, on the other side, the concentrations were decreasing following the order of SA (1.53 mg/m^3^) > IA (1.49 mg/m^3^) > RA (1.38 mg/m^3^) > UA (1.23 mg/m^3^). The SA and IA sites are located at the edge of the city and nearby the outside ring of a highway, therefore, higher traffic emissions of CO might be the main source in SA and IA. At last, for O_3_, the mass loading ranked from high to low as: SA (76.61μg/m^3^) ≈ UA (76.00 μg/m^3^) > IA (70.49 μg/m^3^) > RA (62.88 μg/m^3^). Even though the average mass loading of O_3_ in SA was almost equal to that in UA in all four seasons, the O_3_ in UA was significantly higher than that in SA in summer, indicating a phenomenon that O_3_ pollution has become an increasing concern for the urban residents in Jining. On the other hand, O_3_ in RA was the lowest in all seasons.

Figure 3B illustrates the diurnal pattern of the six standard pollutants in the four functional areas. The different functional areas exhibit very similar diurnal cycles for the same pollutant. Overall, the mass loadings of PM_2.5_ and NO_2_ during night-time were stable but started to drop after 9:00 a.m. After reaching minimum values around 4:00 p.m., they began to increase until 11:00 p.m. For PM_10_, the diurnal pattern is different to PM_2.5_ and exhibits two peaks at 3:00 a.m. and 9:00 a.m. and a valley at 4:00 p.m. Overall, PM and NO_2_ concentrations during night-time surpass daytime values and an obvious decrease appears in the afternoon, which could be interpreted by the strengthened emission (traffic emission, resident heating, etc.) during night-time and an elevated height of the planetary boundary layer (PBL) during the afternoon. Meanwhile, the decreased concentrations of gas pollutants, including SO_2_ and CO, in the afternoon also can be explained by the increased height of PBL, which can dilute those gas pollutants. However, morning peaks (at 9:00 am) of PM, SO_2_ and CO can be attributed to enhanced fossil fuel combustion.

### 3.2. Health risk in four functional areas

**Figure 4:**
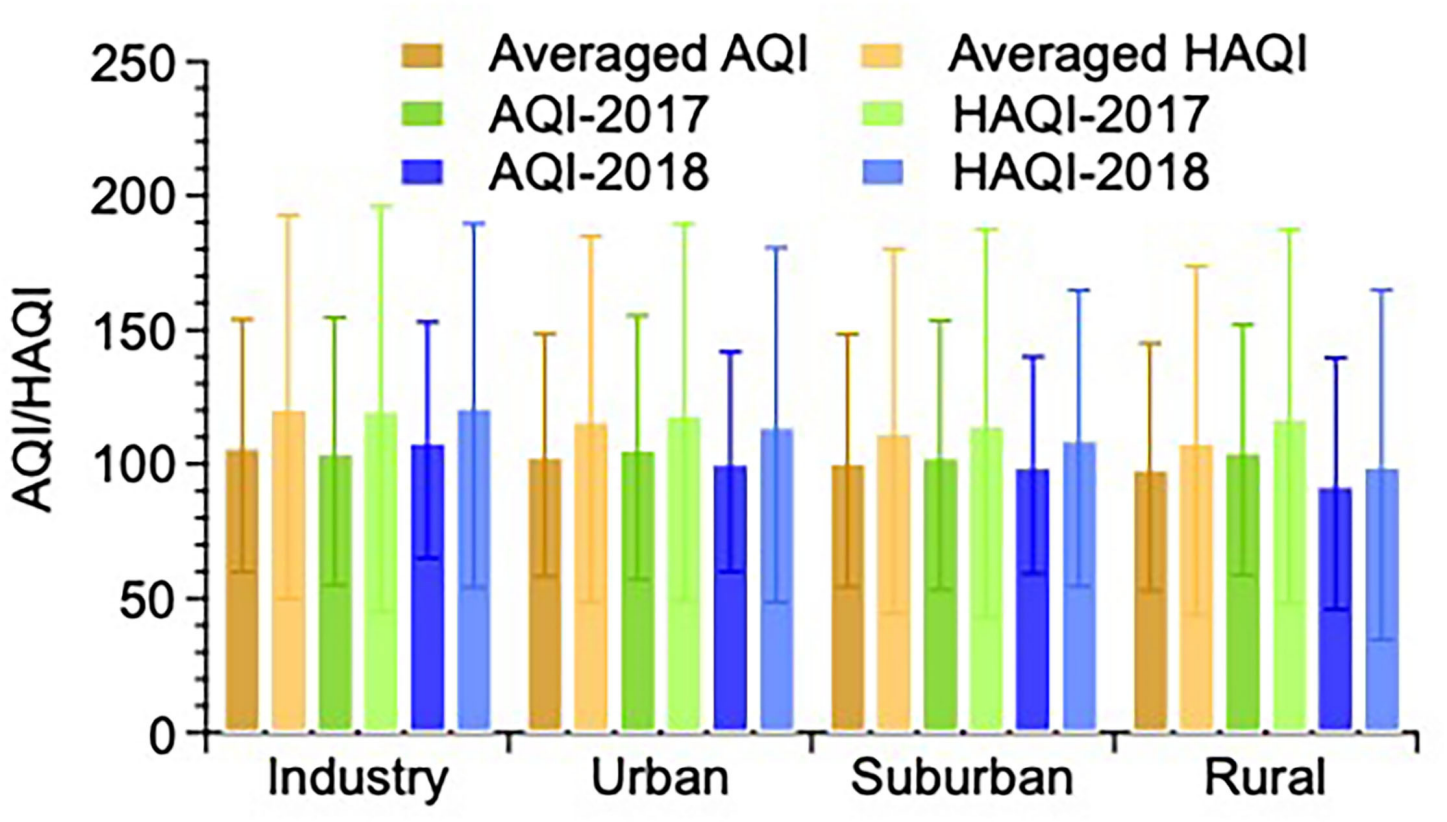
The mean AQI and HAQI average over 2017 and 2018 in four functional areas in Jining.

In the next step, the average AQI and HAQI values were calculated over the 2017–2018 time period based on the daily average values of pollutants (Figure 4). In the four functional areas, the mean value of AQI and HAQI in 2017–2018 decreases following the order: IA (AQI: 106.9 ± 47.0, HAQI: 121.3 ± 71.5) > UA (AQI: 103.6 ± 45.2, HAQI: 117.0 ± 68.2) > SA (AQI: 101.5 ± 47.1, HAQI: 112.5 ± 68.0) > RA (AQI: 99.1 ± 46.1, HAQI: 108.8 ± 65.0). For all functional areas, the mean values of HAQI are higher than the AQI value, which is consistent with the finding of studies concentrated on the comparison between AQI and HAQI (21, 40, 42). The main reason for higher HAQI than AQI is that the HAQI reflects comprehensive health risk rather than the single-pollutant oriented AQI.

**Figure 5:**
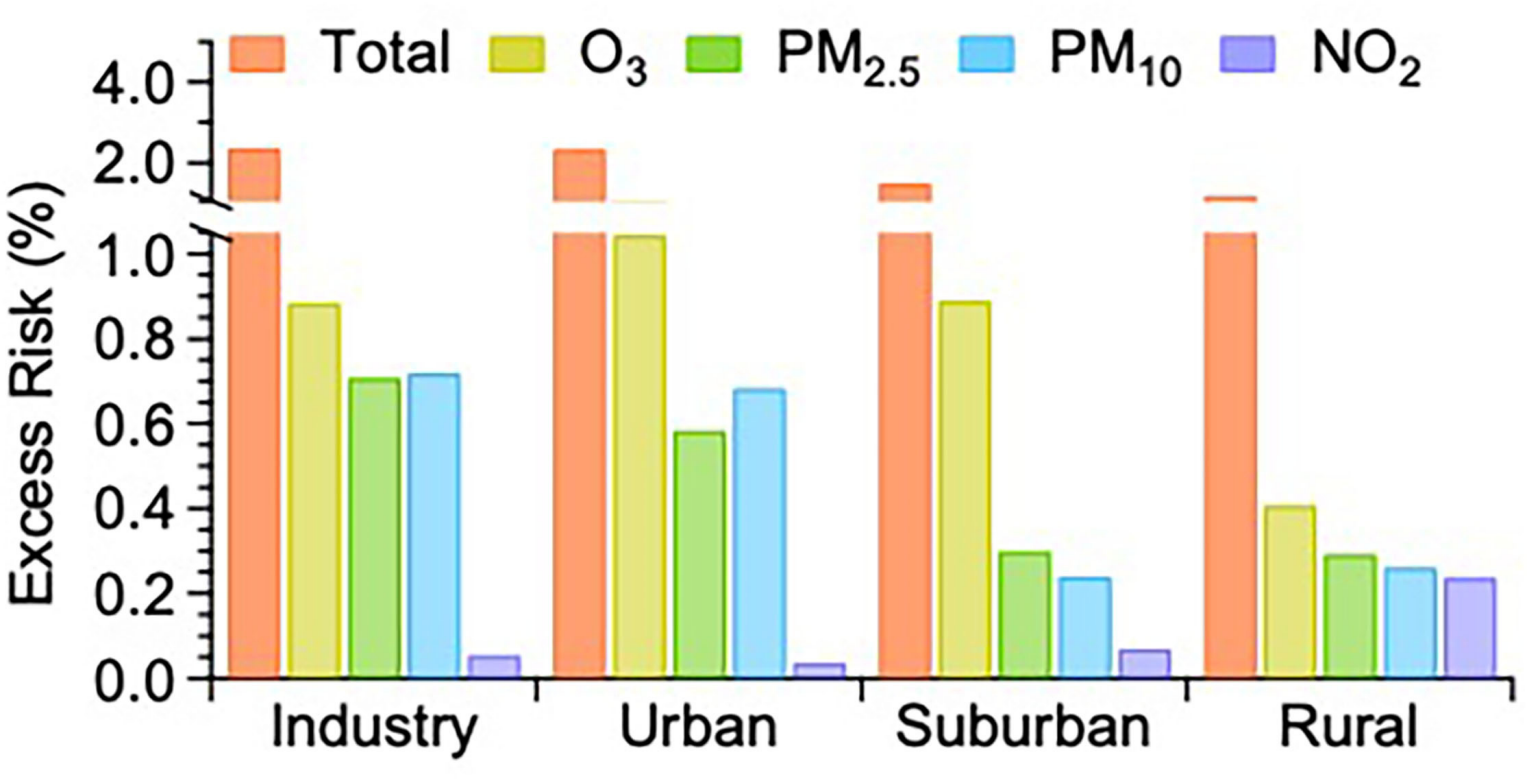
The comparison of excess risks (ERs) averaged over 2017 and 2018 attributable to the sum and individual air pollutants among the four functional areas in Jining.

It is interesting to also look at the total ERs needed as input to the HAQI calculation and which were calculated by using Eq. 3. It should be noted that SO_2_ and CO concentrations were always below the threshold concentration and thus the two pollutants had no exposure health risk to the public people. The total ER in IA (Figure 5) was the highest with a value of 2.38%, followed by 2.35% in UA, 1.50% in SA, and 1.20% in RA, respectively. For total ERs in the four functional areas, ERs of O_3_ (IA: 0.88%, UA: 1.05%, SA: 0.89%, RA: 0.41%) made the dominant contribution to total ERs. For the total ER in IA, the ER of PM_2.5_ and PM_10_ made an almost equal contribution (0.71% and 0.72%) after that of O_3_, followed by the contribution of NO_2_ (0.06%). In UA, the other total ER contributors amounted to 0.58% for PM_2.5_, 0.68% for PM_10_, and 0.04% for NO_2_. In SA, the other three contributions to the total ER were 0.30% for PM_2.5_, 0.24% for PM_10_, and 0.07% for NO_2_, respectively. Except for the ER of O_3_, the ER of PM_2.5_, PM_10_, and NO_2_ in RA were 0.29, 0.26, and 0.24%, respectively. For total ERs in RA, even though the major contributor of O_3_ is rather low compared to the other functional areas, the highest ER for NO_2_ can offset the contribution from O_3_, leading to the not quite low HAQI in RA.

### 3.3. Premature mortality attributable to air pollutants

After evaluating the total ERs from six air pollutants in Jining, we can further investigate the premature mortality attributable to different air pollutants. Based on monitoring data of six pollutants in 2017 and 2018, the all-cause premature mortality by short-term exposure to air pollution in Jining was calculated here. The total premature mortality caused by air pollution for the 2 years was 6,072 and 2,145 for 2017 and 2018, respectively (Table 1). Specifically, the premature mortalities attributable to NO_2_, O_3_, PM_10_, and PM_2.5_ were 912, 1,755, 1,824, 1,581 for 2017 and 175, 666, 593, and 710 for 2018. For the number of premature mortality attributable to PM_2.5_ in 2017, it is almost consistent with the death number of 1,488 in terms of the total population (1.5 million) (60). PM_10_ was the dominant contributor to premature deaths in 2017, but its contribution decreased from 30.0% in 2017 to 27.7% in 2018. The relative contribution of O_3_ increased from 28.9% in 2017 to 31.0% in 2018, exceeding the relative contribution of PM_10_ in 2018. The changing contributions of PM_10_ and O_3_ to the total premature mortality between 2017 and 2018 are directly related to the opposed changes in their observed concentrations. Furthermore, the contribution of NO_2_ decreased from 15.0% in 2017 to 8.2% in 2018. Note, the health effects of SO_2_ and CO are not shown in the table because their concentrations are always under the threshold values and thus do not contribute to premature mortality.

**Table 1:** Premature mortality attributable to short-term exposure to different air pollutants and their emission sources in 2017 and 2018, respectively.

**Air pollutant**	**2017**				**2018**			
	**Premature death (person)**	**Urban**	**Contribution (%)**		**Premature death (person)**	**Urban**	**Contribution (%)**	
		**Suburban**				**Suburban**		
		**Rural**				**Rural**		
		**Industry**				**Industry**		
NO_2_	**912**	17	**15.0**	1.53	**175**	9	**8.2**	1.7
		22		1.7		16		1.4
		392		22.9		34		7.2
		481		24.9		127		15.8
O_3__8h	**1,755**	490	**28.9**	43.5	**666**	236	**31.0**	48.3
		522		40.1		175		47.8
		288		16.8		57		11.8
		456		23.6		198		24.5
PM_10_	**1,824**	298	**30.0**	26.4	**593**	147	**27.7**	30.1
		385		29.6		71		19.5
		528		30.9		184		38.2
		613		31.7		191		23.6
PM_2.5_	**1,581**	322	**26.0**	28.6	**710**	97	**33.1**	19.9
		373		28.6		114		31.2
		501		29.3		206		42.8
		386		19.9		292		36.2
Total	**6,072**	1,127	**100**	100	**2,145**	489	**100**	100
		1,301		100		366		100
		1,708		100		482		100
		1,936		100		808		100

Bold indicate total numbers, rows beside each total from top to bottom are numbers for UA, SA, RA, and IA.

Looking into the four functional areas separately, the number of NO_2_-driven premature deaths in RA and IA was higher in 2017 than that in 2018, and their relative contributions decreased from 22.9% and 24.9% in 2017 to 7.2% and 15.8% in 2018, respectively. Note, the contribution of NO_2_ to premature death was much lower and the inter-annual variation was not significant in the other two regions. Inspection of the contribution of O_3_ to premature death in UA and SA reveals its significance in these areas, notably increasing from 43.5 and 40.1% in 2017 to 48.31 and 47.83% in 2018, respectively, and representing the main factor leading to premature death in UA and SA. In the other two regions, the contribution of O_3_ to premature death was relatively low and the inter-annual variation was not significant.

The contributions of PM_10_ and PM_2.5_ to premature death in the four regions varied between 2017 and 2018. In RA, the contribution rates of PM_10_ and PM_2.5_ increased from 30.9 and 29.3% in 2017 to 38.2 and 42.8% in 2018, respectively. In SA and IA, the contribution rate of PM_10_ decreased from 29.6 and 31.7% in 2017 to 19.5 and 23.6% in 2018, while PM_2.5_ increased from 28.63 and 19.9% in 2017 to 31.2 and 36.2% in 2018, respectively. In RA, the trend of PM_10_ and PM_2.5_ contributions to premature death was thus different to that in the other functional areas.

### 3.4. Identify the contributions of air pollutants to health risk

**Figure 6:**
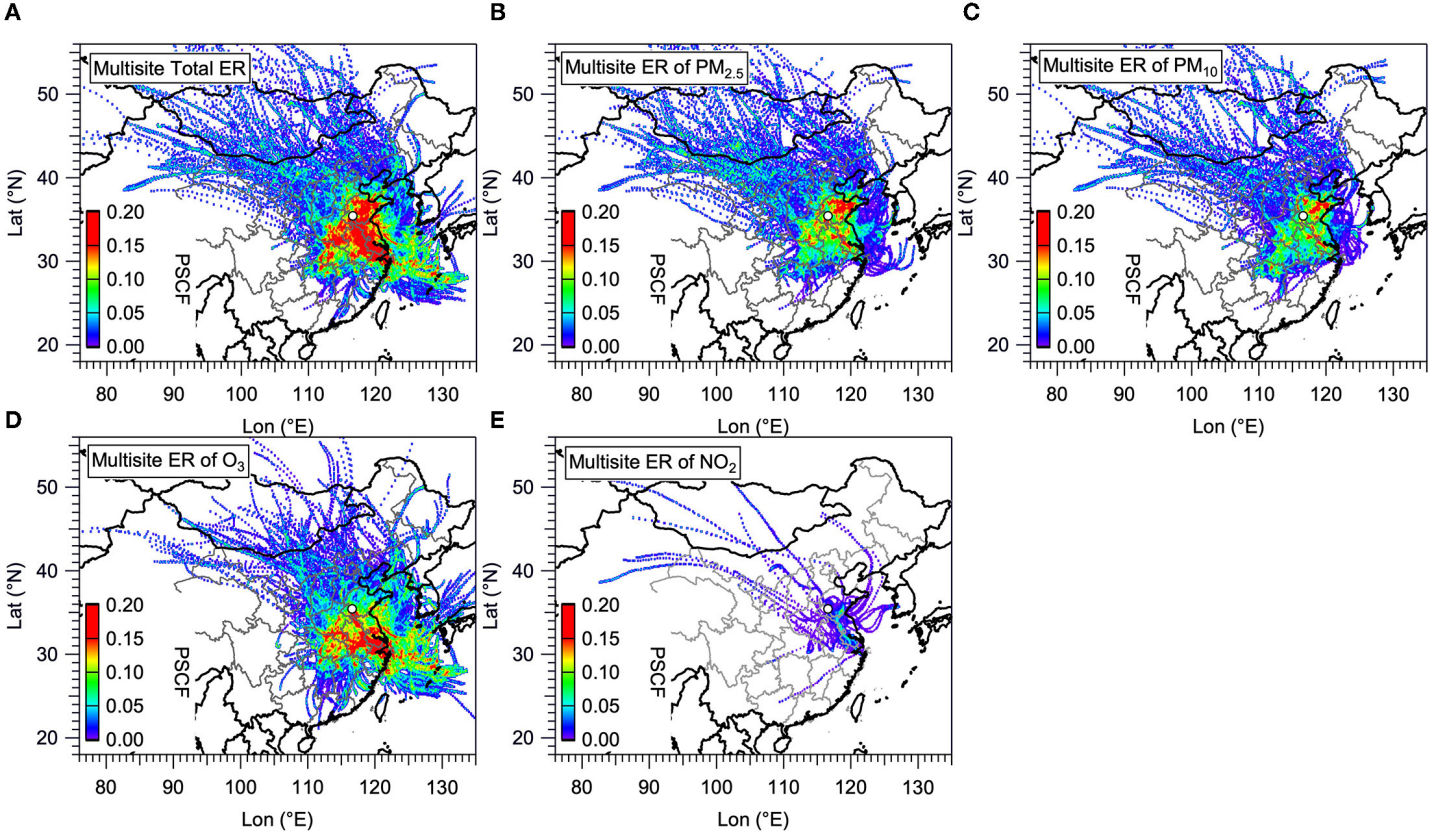
The potential source areas (PSA) of the excess risks (ERs) of air pollutants (based on CAAQS standards) in the four functional areas in Jining estimated based on multi-site emerging method. The color scale represents the possibility of the ER source, while the areas of hot spots covered can be considered as the PSA for each pollutant (**A**: Total; **B**: PM_2.5_; **C**: PM_10_; **D**: O_3_; and **E**: NO_2_).

The PSCF analysis (see methods in section 2.4) was used to identify which functional areas and air pollutants are the major contributors to the health risk in Jining (Figure 6). To this end, the total ER in each functional area is first calculated by adding up the ER of all six pollutants according to Eq. 3, and then the multi-site merging method (see Eq. 17) was applied for calculating the multi-site ER for the total (Figure 6A) and PM_2.5_ (Figure 6B), PM_10_ (Figure 6C), O_3_ (Figure 6D), and NO_2_ (Figure 6E) contributions in Jining, respectively. In Figure 6, the color scale represents the possibility of the ER source, while the areas of hot spots covered can be considered as the Potential Source Areas (PSA) for each pollutant. The information obtained from this analysis is expected to offer important information to the local government in Jining on which air regulation measures to implement to reduce public exposure to health risks depending on the different functional areas.

**Figure 7:**
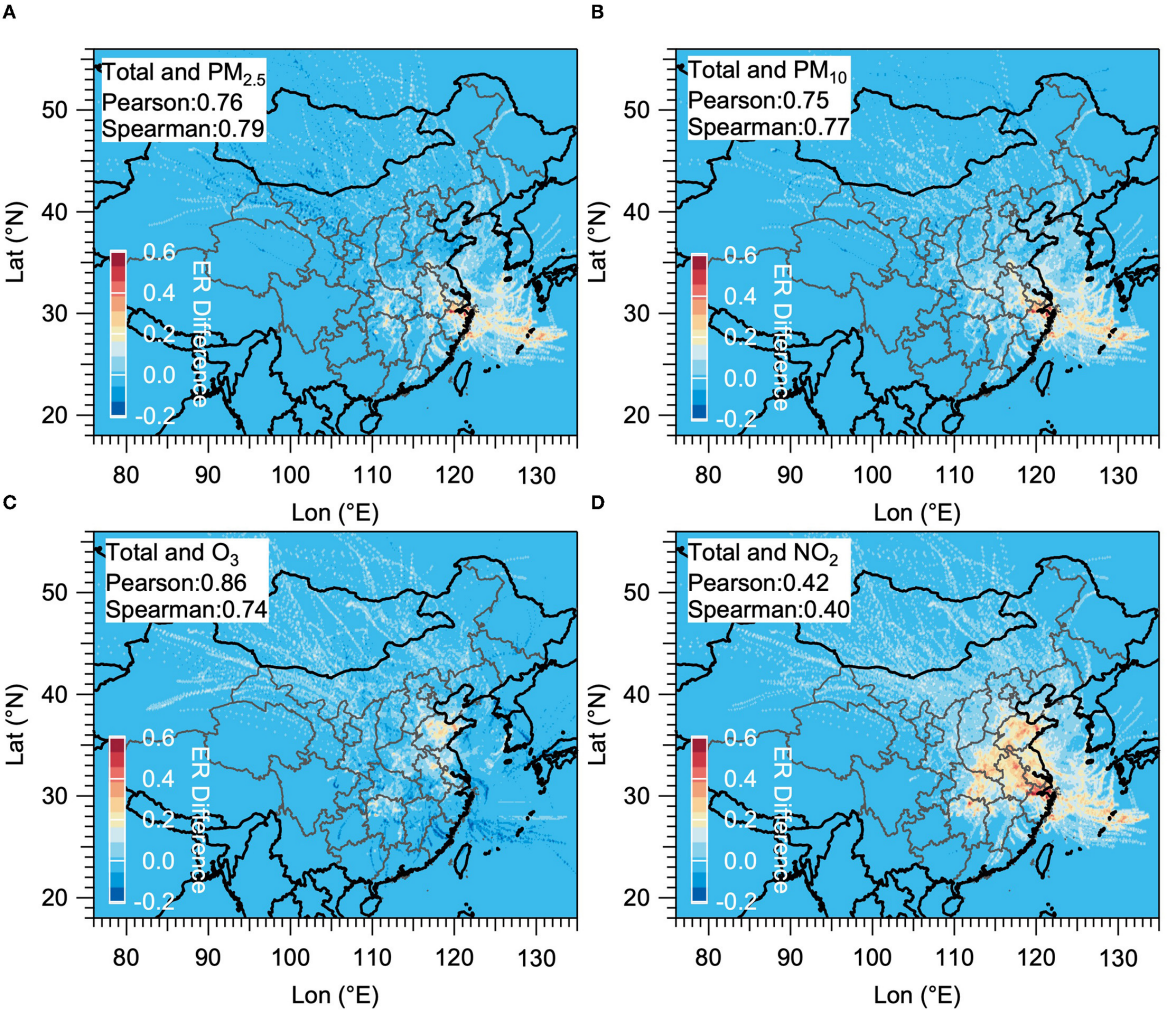
Distribution of the difference between total ER and ER from individual pollutant, and the Pearson coefficient and Spearman coefficient between the two in Jining. The color scale represents the difference between the total ER and ER from each air pollutant, the areas of hot spots covered can be considered as the PSA for each air pollutant (**A**: PM_2.5_; **B**: PM_10_; **C**: O_3_; and **D**: NO_2_).

For the total ER in Jining, the dominant PSA are mainly located in the north and central of Shandong Province, including Jining city itself, and also expand to significant fractions of the southeast of Henan Province and the Anhui Province, and almost the total area of Yangtze River Delta (YRD). Besides, there was still a small part of PSA located in the northwest of Hubei Province and East China Sea extending from Henan Province and YRD, respectively. The hot spot areas in the north direction of ER for PM_2.5_ was larger than that for PM_10_, thus ER for PM_2.5_ was considered as the major contributor of the total ER in the north direction. In the south direction, the contribution to the PSA of the total ER is mostly attributable to O_3_, followed by that attributable to PM_2.5_, PM_10_, and NO_2_. After identifying the PSA of the total ER in different directions, we further calculated the Pearson coefficient (*r*) and Spearman coefficient (*s*) between the PSA for ER of each pollutant and that for the total ER (Figure 7). From the results of the two coefficients, the ER of O_3_ (*r* of 0.86) had the tightest association with the total ER, followed by that of PM_2.5_ (*r* of 0.76), PM_10_ (*r* of 0.75), and NO_2_ (*r* of 0.42) when just considering the *r*. The ER of NO_2_, on the other hand, was only weakly correlated with the total ER exhibiting the lowest *r* of 0.4 and *s* of 0.42. This finding stress that the local government in Jining should take urgent ways to reduce O_3_ pollution as well as PM in the south direction and north direction of Jining City, respectively.

### 3.5. Effects of meteorological factors on health risk

**Figure 8:**
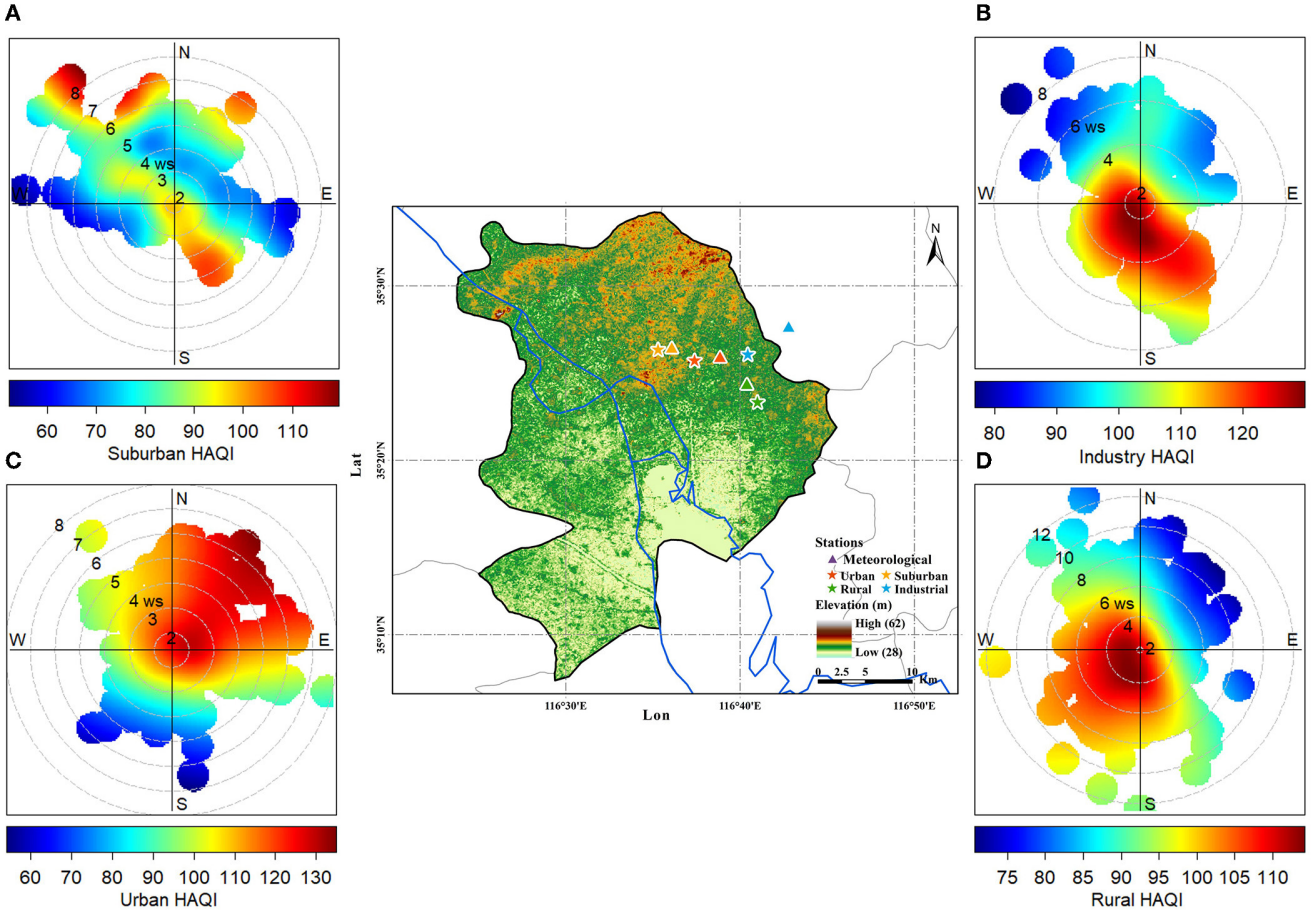
The bivariate polar plots of HAQI in suburban **(A)**, industry **(B)**, urban **(C)**, and rural **(D)** areas in Jining. The horizontal (W–E) and vertical (S–N) axes in the bivariate polar plots represent the wind directions, the length of the radial contours represents the wind speed, and the color bar scale indicates HAQI values.

Figure 8 shows the bivariate polar plots of HAQI in four functional areas during 2017 and 2018. In Figure 8, the horizontal (W–E) and vertical (S–N) axes represent the wind directions, the length of the radial contours represents the wind speed, and the color bar scale indicates HAQI values. HAQI varied depending on the wind speed and wind direction. The layout of Figures 8A–D is displayed according to the actual geographic location of each functional site in Jining. For instance, the UA (Figure 8C) and RA (Figure 8D) sites are located in the west and south of the IA (Figure 8B) site, respectively. The SA (Figure 8A) site is on the west side of the UA. When the wind speed was low in IA and RA, the HAQIs were both higher indicating a local source leading to the high HAQI values. It also reveals high HAQIs for wind directions from the southeast and southwest suggesting two potential transport directions in IA. The RA site also had two potential transport directions in the southwest and northwest. Conversely, when the windspeed was higher at the UA and SA sites, the HAQI resulted in higher values, suggesting high HAQIs at these two sites can be attributed to transport from nearby pollution sources in the northeast and southeast directions. From the analysis above, IA has been identified as a likely source for increased health risk in UA and SA in situations with east wind direction.

**Figure 9:**
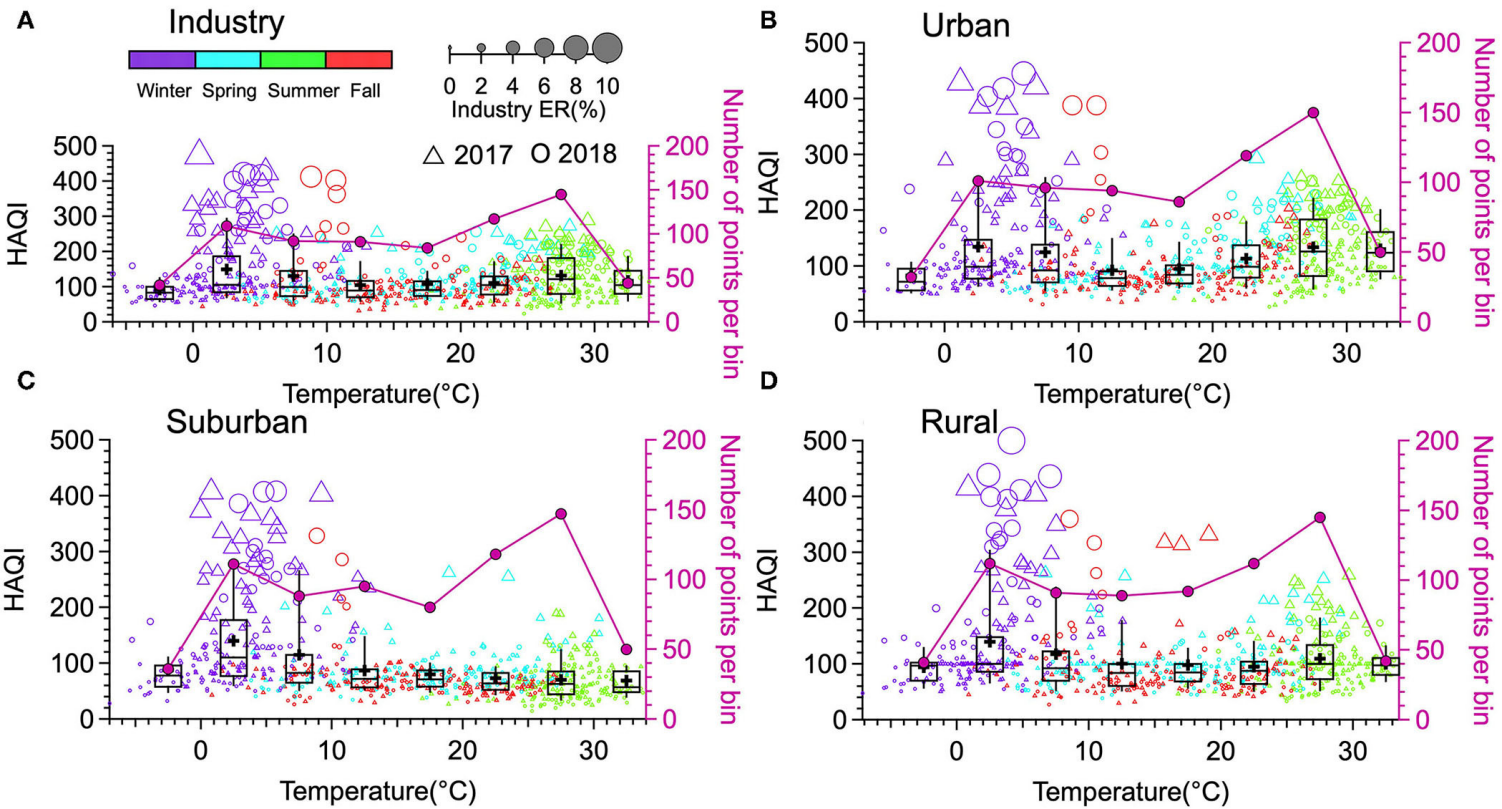
The HAQI variation depends on the temperature in the four functional areas (**A**: Industry; **B**: Urban; **C**: Suburban; **D**: Rural) in Jining. The triangles (HAQI in 2017) and circles (HAQI in 2018) indicate the distribution of HAQI events in each temperature bin, with the circle size depending on the ER values and the circle color indicating the season.

Figure 9 illustrates the HAQI variation depending on the temperature in IA, UA, SA and RA (Figure 5a–d). At each site, the triangles (HAQI in 2017) and circles (HAQI in 2018) indicate the distribution of HAQI events in each temperature bin, with the circle size depending on the ER values and the circle color indicating the season during which the event occurred. Overall, in all four functional areas, more ER days (UA: 150, SA: 147, RA: 145, IA: 145) occurred in the temperature range of 25 to 30°C (that is primarily during summer), but higher averaged HAQI (UA: 134.7, SA: 140.28, RA: 139.66, IA: 149.24) presented in the temperature bin of 0 to 5°C (that is mostly during winter). High frequency of Ozone pollution days led to more ER days in summer, while less PM pollution days coupled with more severe pollution levels attributed to higher average HAQI in winter.

## 4. Policy implication

To better protect the public's health in Jining as well as in the whole of China, the local government should design certain policies and execute mitigation measures to tackle the threat to the public's health. Firstly, the multi-pollutant index should be considered when policymakers are developing relative regulations. Air quality standards are generally constructed based on the summary of the research evidence on the assessment of health impact attributable to each air pollutant separately. With the emergence of the multi-pollutant health index's framework and the increasing epidemiological evidence of the health effects, the future application of the multi-pollutant health index will become possible, even though there are still many uncertainties. Moreover, standards for multi-species air pollution levels should be built. If the multi-pollutant-oriented health risk assessment (including their statistical uncertainty) could be estimated with high reliability, then the air quality standards could be built on the base of the multi-species air pollution level. For example, this study in Jining city found that PM_10_ was the dominant contributor to premature mortality but the O_3_ pollution level increased simultaneously. Thus, it would be better to define a standard for PM_10_ that considers the ozone pollution level. Finally, if the pollution source that leads to health risks for the humans is identified, the mitigation regulations could be designed such that it would account for the relative importance of the primary and secondary pollutants. For example, in Jining city, the ozone pollution level increased from 2017 to 2018, and control measures should be taken that yield a more balances control of the levels of VOC and NOx, which are the precursors of ozone.

## 5. Conclusion and remarks

In this study, four ambient air sampling sites in different functional areas, including urban, suburban, industrial, and rural areas, were selected to explore air pollution characteristics and the exposure health risk to the public in Jining. The spatiotemporal distribution, exposure health risks, and potential source areas of each functional area were compared for 2017 and 2018 in Jining. Overall, all average air pollutant concentrations in Jining decreased between 2017 and 2018, except for O_3_, which showed an increase. The four functional areas showed the same seasonal and diurnal patterns among the six criteria air pollutants considered. The mass concentration of PM and NO_2_ in IA and RA showed higher concentrations, respectively. The total premature deaths attributable to air pollution were 6,072 and 2,145 in 2017 and 2018 respectively, attributing to the decrease of air pollutants' concentrations and reflecting the benefits of controlling air pollution levels to human health in this region. Local pollutant emissions mainly contributed to high HAQI values in IA and RA, while high HAQI in UA and SA may instead be attributed to long-distance pollution transport. The ER of O_3_ was with the highest *r*, reflecting the dominant contributor to the potential source area for total ER in the south, while PM was the main contributor to the potential source area of total ER in the north. Overall, these results highlight that IA is the main local pollution source and that the most urgent measures should be taken to reduce O_3_ pollution and particulate matter (PM), especially in industrial and urban areas to improve public health.

Results demonstrated in this study imply that O_3_ rather than PM might become the primary threat to the public's health and urgent measures should be taken in the IA region in Jining city. However, it should be noted that this health assessment includes uncertainties due to various factors such as the ER calculation, measurement errors, and degree of correction between pollutants etc. More epidemiologic studies are required in the future to validate whether or not the HAQI is reliable to represent the multi-pollutant's health risk. Simultaneously, more attention should be paid on how to select the baseline concentration and ER coefficients since the results are sensitive to these measures as well.

## Data availability statement

The original contributions presented in the study are included in the article/Supplementary material, further inquiries can be directed to the corresponding authors.

## Author contributions

YY: conceptualization, methodology, software, data curation, writing—original draft, software, and validation. XZ, JZ, DN, BW, LW, and MX: data curation. FS: conceptualization, methodology, software, writing—original draft, supervision, software, validation, and writing—review and editing. MH: conceptualization, methodology, supervision, software, validation, and writing—review and editing. All authors contributed to the article and approved the submitted version.
