# Supplementary figures and images for: Characteristics, health risks, and premature mortality attributable to ambient air pollutants in four functional areas in Jining, China
Source: Front Public Health. 2023 Jan 19;11:1075262. doi: 10.3389/fpubh.2023.1075262 (PMC9893643; doi:10.3389/fpubh.2023.1075262)

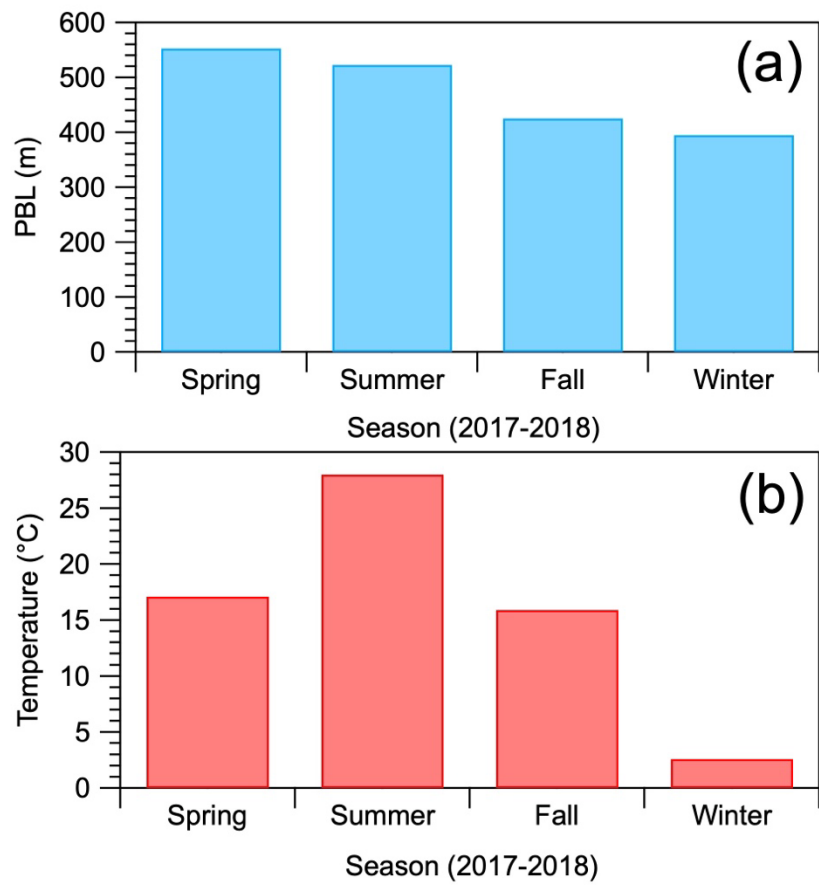

Figure 1. Seasonal distribution of PBL (a) and Temperature (b) averaged over 2017 and 2018 across Jining city

Supplement: Supplementary file 1 [file Image_1.pdf]
