# Supplementary material for: Characteristics, health risks, and premature mortality attributable to ambient air pollutants in four functional areas in Jining, China
Source: Front Public Health. 2023 Jan 19;11:1075262. doi: 10.3389/fpubh.2023.1075262 (PMC9893643; doi:10.3389/fpubh.2023.1075262)
Supplement: Supplementary file 2 [file Table_1.DOCX]

Supplementary Table 1: The reference concentrations for the pollutants in different health categories

|  |  | concentrations (μg/m^3^) | | | | | Category | Health risks |
| --- | --- | --- | --- | --- | --- | --- | --- | --- |
| AQI | PM_2.5_ | PM_10_ | SO_2_ | NO_2_ | CO | O_3_ |  |  |
| 0-50 | 35 | 50 | 50 | 40 | 2 | 100 | Excellent | Satisfactory, no risk |
| 51-100 | 75 | 150 | 150 | 80 | 4 | 160 | Good | Acceptable, may be a moderate risk for a very small number of people |
| 101-150 | 115 | 250 | 475 | 180 | 14 | 215 | Light pollution | Unhealthy for sensitive people (children, older adults, etc.) |
| 151-200 | 150 | 350 | 800 | 280 | 24 | 265 | Moderate pollution | Unhealthy (everyone begins to have adverse health effects) |
| 201-300 | 250 | 420 | 1600 | 565 | 36 | 800 | Serious pollution | Very unhealthy (everyone experience more serious health effects) |
| 301-400 | 350 | 500 | 2100 | 750 | 48 | 1000 | Very severe pollution | Hazardous (healthy people have significant symptoms) |
| 401-500 | 500 | 600 | 2620 | 940 | 60 | 1200 |  |  |
